# Hemodynamic characteristics of high-altitude headache following acute high altitude exposure at 3700 m in young Chinese men

**DOI:** 10.1186/s10194-015-0527-3

**Published:** 2015-05-12

**Authors:** Shi-Zhu Bian, Jun Jin, Qian-Ning Li, Jie Yu, Cai-Fa Tang, Rong-Sheng Rao, Shi-Yong Yu, Xiao-Hui Zhao, Jun Qin, Lan Huang

**Affiliations:** Institute of Cardiovascular Diseases, Xinqiao Hospital, Third Military Medical University, 183 Xinqiao Street, Chongqing, 400037 China; Department of Cardiology, Xinqiao Hospital, Third Military Medical University, Chongqing, China; Department of Neurology, Xinqiao Hospital, Third Military Medical University, Chongqing, China; Department of Ultrasonography, Xinqiao Hospital, Third Military Medical University, Chongqing, China

**Keywords:** Systemic circulation, Cerebral circulation, Hemodynamic characteristics, High-altitude headache, Acute exposure

## Abstract

**Background:**

This study aimed to identify the systemic and cerebral hemodynamic characteristics and their roles in high-altitude headache (HAH) among young Chinese men following acute exposure.

**Methods:**

The subjects (n = 385) were recruited in June and July of 2012. They completed case report form questionnaires, as well as heart rate (HR), blood pressure, echocardiogram and transcranial Doppler examinations at 3700 m following a two-hour plane flight. A subgroup of 129 participants was examined at two altitudes (500 and 3700 m).

**Results:**

HAH was characterized by increased HR and cardiac output (CO) and lower saturation pulse oxygen (SpO_2_) (all p < 0.05). The change in tricuspid regurgitation was also different between the HAH positive (HAH+) and HAH negative (HAH-) subjects. Furthermore, the HAH+ subjects exhibited faster mean (V_m_), systolic (V_s_) and diastolic (V_d_) velocities in the basilar artery (BA; all p < 0.05) and a faster V_d_ ( 25.96 ± 4.97 cm/s vs. 24.76 ± 4.76 cm/s, p = 0.045) in the left vertebral artery (VA). The bilateral VA asymmetry was also significantly different between the two groups. The pulsatility index (PI) and resistance index (RI) of left VA were lower in the HAH subjects (p < 0.05) and were negatively correlated with HAH (p < 0.05). Baseline CO and V_m_ in left VA (or right MCA in different regressions) were independent predictors for HAH, whereas CO/HR and ΔV_d_ (V_d_ difference between bilateral VAs) were independent risk factors for HAH at 3700 m.

**Conclusions:**

HAH was characterized, in part, by increased systemic hemodynamics and posterior cerebral circulation, which was reflected by the BA and left VA velocities, and lower arterial resistance and compliance. Furthermore, baseline CO and V_m_ in left VA or right MCA at sea level were independent predictors for HAH, whilst bilateral VA asymmetry may contribute to the development of HAH at high altitude.

**Electronic supplementary material:**

The online version of this article (doi:10.1186/s10194-015-0527-3) contains supplementary material, which is available to authorized users.

## Background

High-altitude headache (HAH) is the primary critical complaint after high altitude exposure [1]. It is defined as a headache that occurs within 24 hours after ascending to 2500 m or higher [2]. HAH affects tourists’ and labor workers’ daily life and work due to its high incidence and discomfort experience [3]. HAH has also been defined as the primary symptom in diagnosis of acute mountain sickness [4].

Over the past several decades, epidemiology, clinical characteristics, risk factors, prevention and treatment aspects of HAH have been systematically studied in mountaineers [5, 2, 6, 7]. Clinical characteristics studies have showed that HAH presents as a sudden attack of migraine that is accompanied by nausea [6]. Previous studies have demonstrated that increased heart rate (HR) and self-anxiety scores, lower saturation pulse oxygen (SpO_2_) and a history of primary headache are all independent risk factors for HAH [8, 9]. Though oxygen inhalation, acetaminophen and aspirin have been used to treat HAH, the effects are quite often inconsistent [10, 11]. Despite accumulating evidence regarding HAH and related issues, the underlying mechanisms remain elusive [12, 13, 1].

HAH exhibits an impulsivity property [14], which may be related to the hemodynamics (or blood flow in artery) of patients. While most previous studies have focused on the risk factors and epidemiology of HAH, few of them give proper attentions to the hemodynamic characteristics of HAH [8, 9, 7]. Furthermore, most of these studies have been conducted in mountaineers, which may not reflect representative properties of HAH [5, 9, 12].

To the best of our knowledge, the hemodynamic characteristics of and their roles in HAH has not been fully uncovered. Thus, we postulate that hemodynamics especially cerebral hemodynamic parameters are related HAH and they may be predictors for HAH. Therefore, we performed this large sample size cohort study to explore the hemodynamic characteristics of HAH via examinations of the systemic and cerebral hemodynamic parameters using echocardiogram and transcranial Doppler sonography, respectively, following acute high altitude exposure (within 24 h after arrival at 3700 m) to facilitate our understanding of the disease process and provide insight into the specific phenotypes associated with the disease course.

## Methods

### Participants and procedures

#### Participants

Three hundred eighty-five participants were recruited in June and July of 2012 according to specific inclusion and exclusion criteria. The inclusion criteria were as follows: healthy males between the ages of 18 and 60 years who had no high-altitude exposure in recent two years. Individuals with any of the following conditions were excluded: neuropsychosis, cerebrovascular diseases, cardiovascular diseases, respiratory diseases, malignant tumors and liver or kidney disorders.

The study was reviewed and approved by the Ethics Committee of Xinqiao Hospital of Third Military Medical University. The study was thoroughly explained to all subjects who agreed to participate, and all volunteers signed informed consent forms prior to study participation.

#### Procedures

The subjects were transported to an altitude of 3700 m by plane over two hours of travel in June and July of 2012. None of the subjects drinks coffee regularly. The coffee, tea, and other caffeine-containing drinks as well as alcohol were avoided before the examinations. Baseline measurements of systemic and cerebral hemodynamic parameters were examined one week prior to the departure at sea level (Chengdu, 500 m) in a subgroup of 129 participants. The participants will receive regular training after a three days’ rest. They did the daily life activities and avoided any heavy exercises or physical labor in the first 72 hours at 3700 m. The field trials were performed within 18 to 24 hours after their arrivals at 3700 m. Structured case report questionnaires were used to record the subjects’ demographic information (i.e., age, body mass index (BMI), smoking and alcohol consumption), primary headache history [None of them takes medication regularly due to primary headache. However, severe headache (HAH) subjects were treated with NSAIDs or Sanlietong (Taiji medicine, Xi’an, China) as necessary at 3700 m] and HAH symptoms (0 = no headache; 1 = mild headache; 2 = moderate headache; 3 = severe headache).

### Measurements of systemic hemodynamic parameters

The subjects’ systolic blood pressure (SBP), diastolic blood pressure (DBP), HR and SpO_2_ were measured using a sphygmomanometer (HEM-6200, OMRON, China) and a pulse oximeter (NONIN-9550, Nonin Onyx, USA) after the subjects had sat at rest for 30 min. Each subject received an echocardiogram examination (ultrasonography system, CX50, Philips, USA) that involved measurements of the end-diastolic internal diameters of the left atrium (LA), left ventricle (LV), right atrium (RA), right ventricle (RV) and pulmonary artery (PA), in addition to the stroke volume (SV), cardiac output (CO) and ejection fraction (EF).

### Measurements of cerebral hemodynamic parameters

The examinations at both altitudes were performed in the morning after an overnight fast and caffeine containing drinks were avoided.

Transcranial Doppler sonography examinations were performed by the same technician using an ultrasonography system with a 2 Hz probe (EME TC2021-III, NICOLET, USA). For each subject, the following parameters were recorded: the mean velocity (V_m_), systolic velocity (V_s_), diastolic velocity (V_d_), pulsatility index (PI) and resistance index (RI). These measurements were taken in the bilateral middle cerebral artery (MCA) for anterior circulation and in the vertebral artery (VA) and basilar artery (BA) for posterior circulation.

The asymmetry of the MCA and VA was calculated as ΔV = left velocity - right velocity for each lateral MCA or VA, whereas the asymmetry index (AI) was calculated as AI = ΔV/ [(left Vm + right Vm)/2]. The cerebrovascular conductance index was calculated as the V_m_MCA_/mean arterial blood pressure.

#### Statistical analysis

The case report forms were excluded if the demographic information was not completed, the velocity could not be identified at the depths previously described or the systemic hemodynamic parameters were not measured.

The normally distributed measurement variables (age, BMI, SBP, DBP, SpO_2_, HR, echocardiography parameters and velocities, PIs and RIs of the BA, MCAs and VAs) were expressed as the mean ± standard deviation (SD). The non-normally distributed variables were presented as the medians (interquartile range). The enumerated data are expressed as the rate of occurrence (%).

**Fig. 1:**
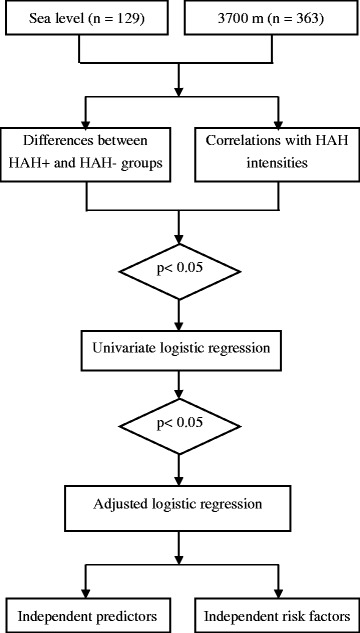
The fluidogram of the statistical analysis

The normally distributed variables for the systemic and cerebral circulation measurements were compared using a paired Student’s t-test between the 500 and 3700 m altitudes and were analyzed using an independent samples t-test between the HAH positive (HAH+) and HAH negative (HAH-) groups at both altitudes. The asymmetries, AIs and tricuspid regurgitation measurements were compared using a Mann–Whitney U test. The relationships between HAH and the previously described parameters at 500 m and 3700 m were analyzed using Spearman’s correlations. Variables with a p < 0.05 in correlation analyses or significant difference between HAH+ and HAH- groups were included in univariate logistic regression. Adjusted logistic analyses were used to identify independent predictors or risk factors for HAH after univariate logistic regression (Fig 1). Variables with colinearity were analyzed in different models (adjusted by primary variables or calculated variables respectively) to reduce the bias.

The statistical analyses were performed using SPSS 19.0 software for Windows. P ≤ 0.05 was considered statistically significant. Statisticians from Third Military Medical University were consulted regarding the statistical methods and results.

## Results

### Clinical characteristics of the subjects

Three hundred eighty-five subjects received both echocardiography and transcranial Doppler examinations at 3700 m of elevation (363 subjects had completely valid transcranial Doppler and echocardiography parameters). Baseline measurements at 500 m were also recorded among 129 individuals. The subjects in this study were characterized by a mean age of 23.35 ± 4.37 years and a mean BMI of 21.81 ± 2.50 kg/m^2^. The ethnicity of the population was primarily Han Chinese (83.2%). Overall, in our study, the incidence of HAH following acute exposure to 3700 m was 75.2% (273 of 363).

### Alterations in systemic and cerebral hemodynamics

Both systemic and cerebral circulations were dramatically altered following acute exposure to high altitude from sea level. However, the blood pressure parameters, including the SBP, DBP, pulse pressure and mean arterial blood pressure, did not significantly increase after the acute exposure. The SpO_2_ decreased from 98.34 ± 1.06% to 88.72 ± 2.71% (p < 0.001). The LA, RA and RV all significantly decreased (all p < 0.001). However, the EF and CO significantly increased, as well as the velocity in the bilateral MCAs. In VAs and BA, the V_d_ exhibited a significant increase. However, the VAs asymmetries did not dramatically change. The changes in the hemodynamic parameters are summarized in Table 1.

**Table 1:** Alterations in systemic and cerebral hemodynamics (N = 129)

	Sea level	3700 m	p value
Systemic hemodynamics			
SBP	116.32 ± 15.24	115.84 ± 11.84	0.782
DBP	74.65 ± 10.93	76.24 ± 9.40	0.156
ΔBP	41.67 ± 11.71	39.61 ± 8.05	0.131
MAP	88.54 ± 11.25	89.44 ± 9.55	0.444
HR	63.58 ± 10.19	83.98 ± 11.90	<0.001**
SpO_2_	98.34 ± 1.06	88.72 ± 2.71	<0.001**
LA	30.91 ± 1.73	30.00 ± 1.92	<0.001**
LV	46.47 ± 2.45	46.15 ± 2.04	0.184
RA	35.70 ± 2.23	34.21 ± 2.15	<0.001**
RV	35.49 ± 2.08	33.72 ± 2.42	<0.001**
PA	20.24 ± 1.60	19.92 ± 1.60	0.097
EF	62.94 ± 5.49	66.56 ± 3.92	<0.001**
SV	65.24 ± 12.22	67.30 ± 8.31	0.088
CO	4.16 ± 0.95	5.32 ± 1.09	<0.001**
Tricuspid regurgitation	0.500 (1.00)	0.500(0.500)	0.885
Cerebral hemodynamics			
Left side MCA			
V_m_	62.08 ± 10.39	65.79 ± 11.78	<0.001**
V_s_	95.95 ± 14.60	99.80 ± 15.93	0.002**
V_d_	42.69 ± 7.91	46.47 ± 9.07	<0.001**
PI	0.867 ± 0.133	0.817 ± 0.133	<0.001**
RI	0.554 ± 0.048	0.534 ± 0.048	<0.001**
Left side VA			
V_m__	32.77 ± 5.14	35.20 ± 5.83	<0.001**
V_s__	50.70 ± 7.50	51.57 ± 7.91	0.233
V_d__	21.91 ± 4.095	24.82 ± 4.86	<0.001**
PI	0.895 ± 0.181	0.770 ± 0.168	<0.001**
RI	0.570 ± 0.067	0.516 ± 0.061	<0.001**
Right side MCA			
V_m_	58.79 ± 10.18	63.25 ± 11.58	<0.001**
V_s_	90.50 ± 14.18	94.71 ± 15.01	<0.001**
V_d_	40.35 ± 7.49	44.55 ± 9.04	<0.001**
PI	0.864 ± 0.150	0.804 ± 0.130	<0.001**
RI	0.556 ± 0.055	0.530 ± 0.050	0.293
Right side VA			
V_m_	34.33 ± 4.77	36.47 ± 5.73	<0.001**
V_s_	50.60 ± 6.55	51.78 ± 7.83	0.058
V_d_	23.24 ± 4.12	25.90 ± 4.55	<0.001**
PI	0.808 ± 0.162	0.715 ± 0.117	<0.001**
RI	0.539 ± 0.061	0.499 ± 0.048	<0.001**
BA			
V_m_	39.66 ± 7.75	41.85 ± 9.58	0.002**
V_s_	67.93 ± 13.57	66.19 ± 13.46	0.117
V_d_	25.07 ± 5.85	28.59 ± 7.71	<0.001**
PI	1.093 ± 0.251	0.920 ± 0.220	<0.001**
RI	0.627 ± 0.068	0.567 ± 0.073	<0.001**
Asymmetry of the bilateral VAs			
ΔV_s_	0.0 (11.0)	0.0 (12.0)	0.645
ΔV_d_	−1.0 (6.0)	−1.0 (6.0)	0.623
ΔV_m_	−1.0 (7.0)	−2.0 (8.0)	0.529
AI%	−3.5 (20.2)	−2.9 (18.1)	0.190
Conduction			
CVCi	0.695 ± 0.145	0.730 ± 0.149	0.011*

BA: basilar artery; CO: cardiac output; CVCi : cerebrovascular conductance index; DBP: diastolic blood pressure; EF: ejection fraction; HAH: high-altitude headache; HAH+: HAH positive or with HAH; HAH-: HAH negative or without HAH;HR: heart rate; LA: end-diastolic internal diameters of left atrium; LV: end-diastolic internal diameters of left ventricle; MCA: middle cerebral artery; PA: end-diastolic internal diameters of pulmonary artery; PI: pulsatility index; RA: end-diastolic internal diameters of right atrium; RI: resistance index; RV: end-diastolic internal diameters of right ventricle; SBP: systolic blood pressure;SpO2: saturation pulse oxygen; SV: stroke volume; VA: vertebral artery; Vs: systolic velocity; Vd: diastolic velocity; Vm: mean velocity;ΔVs: difference in systolic velocity between bilateral isonym cerebral arteries; ΔVd: difference in diastolic velocity between bilateral isonym cerebral arteries;ΔVm: Difference in mean velocity between bilateral isonym cerebral arteries. Cerebrovascular conductance index (CVCi) = V_m_MCA_/MAP

#: N = 129;

*p is 0.05 or less, **p is 0.01 or less

Most of the cerebral hemodynamic parameters altered dramatically from sea level to 3700 m and systemic hemodynamic parameters altered dramatically in part

### Clinical and hemodynamic characteristics of HAH at 3700 m

Only age (p = 0.044) was significantly higher in the HAH+ group as compared with the HAH- group regarding demographic data. The HAH+ patients were characterized by increased HR (87.96 ± 13.02 vs. 84.41 ± 13.11 beats per min, p = 0.026) and decreased SpO_2_ (87.94 ± 3.19 vs. 88.70 ± 2.98%, p = 0.048). Regarding blood pressure, there was no significant difference between the HAH+ and HAH- groups in the SBP, DBP, pulse pressure or mean arterial blood pressure. Although the LA, LV, RA, RV, PA, EF, SV and tricuspid regurgitation were not significantly different in the HAH+ subjects compared with the HAH- subjects (all p > 0.05), the CO was significantly increased in the HAH+ group (p = 0.001).

Anterior circulation, which was assessed via measurements of velocities in MCAs, was similar in the HAH+ and HAH- groups in bilateral MCAs (all p > 0.05). The PIs and RIs in the right and left MCA were not different between the two groups. For posterior circulation, the velocities in BA, including V_s_ (65.46 ± 11.54 vs. 68.81 ± 14.84, p = 0.028), V_d_ (28.61 ± 7.46 vs. 30.47 ± 7.52, p = 0.042) and V_m_ (41.42 ± 8.64 vs. 44.18 ± 9.74, p = 0.017), were significantly different in the HAH- group as compared with the HAH+ group, respectively. Similar to the MCA, the PI (p = 0.151) and RI (p = 0.292) of the BA were not significantly different between the HAH+ and HAH- groups. Furthermore, the V_d_ in left VA was significantly increased in the HAH+ group compared with the HAH- group (25.96 ± 4.97 vs. 24.76 ± 4.76 cm/s, p = 0.045), whereas the V_s_ and V_m_ were not significantly different between the two groups. Additionally, the HAH+ patients also exhibited a lower RI and PI in the left VA (p = 0.040 and 0.025, respectively). However, in the right VA, the velocities and pulsate indexes showed no differences between HAH+ group and HAH- one (all p > 0.05, Table 2).

**Table 2:** Differences between HAH+ and HAH- groups in demographics, systemic and cerebral hemodynamics (N = 363)

Headache^a^	HAH-	HAH+	HAH intensities			p (H+ vs. H-)
Cases(n)	(90)	(273)	Mild (215)	Moderate (52)	Sever (6)	
Demographic factors						
Age	22.59 ± 3.95	23.60 ± 4.48	23.12 ± 4.26	25.47 ± 4.99	25.00 ± 3.69	0.044*
BMI	21.52 ± 2.11	21.90 ± 2.61	21.85 ± 2.55	21.96 ± 2.89	23.56 ± 2.09	0.199
Smoking(yes)	20(22.2%)	66(24.2%)	52(24.2%)	11(21.2%)	3(50.0%)	0.705
Alcohol consumption(yes)	54(60.0%)	159(58.2%)	126 (58.6%)	28(53.8%)	5(83.3%)	0.796
Systemic hemodynamics						
SBP	117.28 ± 11.62	118.66 ± 12.03	118.84 ± 12.38	118.17 ± 11.04	116.67 ± 7.76	0.340
DBP	77.52 ± 10.83	78.86 ± 9.97	78.89 ± 10.21	78.92 ± 9.26	77.33 ± 8.43	0.279
ΔBP	39.76 ± 7.14	39.80 ± 6.59	39.94 ± 7.04	39.25 ± 4.54	39.33 ± 5.24	0.958
MAP	90.77 ± 10.57	92.13 ± 10.24	92.21 ± 10.47	92.01 ± 9.65	90.44 ± 7.83	0.280
HR	84.41 ± 13.11	87.96 ± 13.02	87.35 ± 12.65	89.35 ± 13.26	98.00 ± 20.66	0.026*
SpO_2_	88.70 ± 2.98	87.94 ± 3.19	88.16 ± 3.01	87.06 ± 3.81	87.83 ± 3.06	0.048*
LA	29.82 ± 2.12	29.70 ± 2.18	29.75 ± 1.99	29.40 ± 2.84	30.33 ± 2.50	0.632
LV	45.78 ± 2.27	46.00 ± 2.25	46.04 ± 2.21	45.85 ± 2.44	45.83 ± 2.32	0.425
RA	33.98 ± 2.04	34.10 ± 2.32	34.14 ± 2.27	34.06 ± 2.56	33.17 ± 2.32	0.661
RV	33.48 ± 2.24	33.70 ± 2.56	33.78 ± 2.56	33.50 ± 2.60	32.50 ± 2.07	0.469
PA	19.83 ± 1.14	19.95 ± 1.42	19.93 ± 1.42	19.98 ± 1.48	20.33 ± 1.03	0.452
EF	66.38 ± 4.28	67.16 ± 4.72	67.29 ± 4.82	66.87 ± 4.40	64.83 ± 3.31	0.165
SV	65.92 ± 8.96	67.32 ± 9.72	67.57 ± 9.79	66.66 ± 9.42	64.17 ± 10.63	0.230
CO	5.16 ± 1.14	5.60 ± 1.02	5.60 ± 1.03	5.65 ± 1.02	5.60 ± .663	0.001**
Tricuspid regurgitation	0.50(0.80)	0.50(0.60)	0.50(0.60)	0.50(0.80)	0.40(0.68)	0.135
Cerebral hemodynamics						
Left side MCA						
V_m_	67.46 ± 12.18	67.41 ± 11.74	66.91 ± 10.87	69.35 ± 14.87	68.50 ± 11.47	0.973
V_s_	102.17 ± 14.94	101.41 ± 15.91	100.79 ± 15.15	103.92 ± 18.83	102.00 ± 15.82	0.693
V_d_	48.0 ± 10.12	47.96 ± 9.10	47.51 ± 8.37	49.46 ± 11.67	51.00 ± 8.60	0.964
PI	0.820 ± 0.169	0.800 ± 0.130	0.802 ± 0.127	0.799 ± 0.141	0.750 ± 0.130	0.260
RI	0.531 ± 0.063	0.527 ± 0.0490	0.528 ± 0.048	0.526 ± 0.051	0.499 ± 0.057	0.532
Left side VA						
V_m_	35.18 ± 5.57	36.38 ± 6.19	35.93 ± 5.66	37.63 ± 6.59	41.67 ± 14.40	0.103
V_s_	51.28 ± 7.05	52.16 ± 8.49	51.75 ± 7.93	53.19 ± 9.20	57.83 ± 17.59	0.374
V_d_	24.76 ± 4.76	25.96 ± 4.97	25.46 ± 4.49	27.35 ± 5.08	32.00 ± 11.93	0.045*
PI	0.765 ± 0.159	0.727 ± 0.153	0.738 ± 0.153	0.690 ± 0.150	0.640 ± 0.102	0.040*
RI	0.517 ± 0.065	0.500 ± 0.061	0.505 ± 0.058	0.482 ± 0.070	0.454 ± 0.053	0.025*
Right side MCA						
V_m_	63.51 ± 13.67	64.46 ± 11.45	64.24 ± 11.26	64.87 ± 11.57	68.67 ± 17.61	0.518
V_s_	95.59 ± 17.61	96.24 ± 15.67	96.17 ± 15.55	96.13 ± 15.65	99.83 ± 22.29	0.740
V_d_	45.11 ± 10.96	45.75 ± 8.73	45.53 ± 8.52	46.12 ± 8.95	50.67 ± 13.74	0.573
PI	0.812 ± 0.144	0.792 ± 0.124	0.796 ± 0.121	0.780 ± 0.125	0.734 ± 0.190	0.191
RI	0.530 ± 0.053	0.526 ± 0.051	0.527 ± 0.051	0.521 ± 0.047	0.493 ± 0.076	0.440
Right side VA						
V_m_	37.27 ± 6.18	37.27 ± 6.02	37.00 ± 5.80	38.63 ± 6.89	35.17 ± 4.66	0.991
V_s_	52.51 ± 7.69	52.26 ± 8.29	52.11 ± 8.09	53.42 ± 9.21	47.50 ± 6.06	0.800
V_d_	26.72 ± 5.09	26.55 ± 4.74	26.26 ± 4.56	27.75 ± 5.33	26.33 ± 4.89	0.764
PI	0.716 ± 0.208	0.693 ± 0.117	0.702 ± 0.117	0.669 ± .114	0.606 ± 0.056	0.202
RI	0.500 ± 0.119	0.491 ± 0.053	0.494 ± 0.052	0.480 ± .053	0.448 ± 0.037	0.317
BA						
V_m_	41.42 ± 8.64	44.18 ± 9.74	43.80 ± 9.42	46.33 ± 10.91	38.83 ± 8.06	0.017*
V_s_	65.46 ± 11.54	68.81 ± 14.84	68.60 ± 14.32	70.81 ± 16.13	59.17 ± 19.63	0.028*
V_d_	28.61 ± 7.46	30.47 ± 7.52	30.07 ± 7.31	32.29 ± 8.31	28.83 ± 6.27	0.042*
PI	0.914 ± 0.224	0.879 ± 0.193	0.890 ± 0.192	0.844 ± 0.183	0.760 ± .262	0.151
RI	0.564 ± 0.078	0.554 ± 0.068	0.559 ± 0.066	0.542 ± 0.069	0.496 ± .086	0.292
Asymmetry of the bilateral VAs						
ΔV_s_	−1.00 (8.00)	0. 00 (9.00)	0.00(9.00)	0.00 (10.50)	5.00 (28.5)	0.126
ΔV_d_	−3.00 (6.25)	−1.00 (6.00)	−1.00(7.00)	−1.00 (3.75)	2.00 (18.5)	0.010*
ΔV_m_	−2.50 (8.00)	−1.00 (6.00)	−1.00(6.00)	−1.00 (4.75)	1.00 (23.75)	0.046*
AI%	−6.6(22.0)	−2.7 (18.0)	−2.9(18.4)	−2.1(13.8)	2.8 (52.2)	0.040*
Conduction						
CVCi	0.729 ± 0.145	0.724 ± 0.140	0.719 ± 0.135	0.737 ± 0.153	0.767 ± 0.191	0.758

*p is 0.05 or less, **p is 0.01 or less

HAH was characterized with higher velocity in posterior cerebral circulation and higher HR but lower SpO_2_

The asymmetry of the bilateral VAs, including the ΔV_d_ (p = 0.010), ΔV_m_ (p = 0.046) and AI (p = 0.040), in the HAH patients was significantly different than the HAH- subjects. The cerebrovascular conductance index (0.724 ± 0.140 vs.0.729 ± 0.145, respectively, p = 0.758) was similar in the HAH+ and HAH- subjects (Table 2).

The Spearman’s correlation analyses demonstrated that age (r = 0.178, p = 0.001), HR (r = 0.143, p = 0.006) and CO (r = 0.175, p = 0.001) exhibited significantly positive relationships with the HAH severity, whereas the SpO_2_ (r = −0.148, p = 0.005) exhibited an inverse relationship. The other parameters in systemic hemodynamics were not closely associated with HAH (all p > 0.05). The anterior circulation parameters, including the velocities, PIs and RIs in the bilateral MCA, had no associations with HAH severity. With the exception of the V_s_, the other parameters (V_m_, V_d_, PI and RI) of the BA exhibited significant associations with HAH (p was 0.023, 0.021, 0.045 and 0.042, respectively). On the left side VA, the V_d_ (r = 0.157, p = 0.003) and V_m_ (r = 0.120, p = 0.022) exhibited positive relationships with HAH, whereas the PI (r = −0.152, p = 0.004) and RI (r = −0.166, p = 0.001) were negatively correlated with HAH. The parameters on the right side of the VA were not as closely correlated with HAH. Furthermore, associations between VA asymmetries and HAH were also identified in the bilateral VAs: ΔV of V_d_ (r = 0.137, p = 0.009), ΔV of V_m_ (r = 0.105, p = 0.046) and AI (r = 0.106, p = 0.043). The relationships between HAH and the hemodynamic parameters are shown in Table 3.

**Table 3:** Relationships between HAH and demographic, systemic and cerebral hemodynamic parameters (N = 363)

	Headache score				
	r	p		r	p
Demographic factors			PI	−0.152	0.004**
Age	0.178	0.001**	RI	−0.166	0.001**
BMI	0.075	0.155			
			Right side MCA		
Systemic hemodynamics			V_m_	0.078	0.136
SBP	0.031	0.561	V_s_	0.055	0.297
DBP	0.031	0.560	V_d_	0.086	0.103
ΔBP	0.009	0.865	PI	−0.099	0.059
MAP	0.033	0.535	RI	−0.087	0.097
HR	0.143	0.006**			
SpO_2_	−0.148	0.005**	Right side VA		
LA	−0.035	0.507	V_m_	0.039	0.455
LV	0.027	0.610	V_s_	<0.001	0.997
RA	0.010	0.851	V_d_	0.036	0.499
RV	0.001	0.992	PI	−0.083	0.115
PA	0.058	0.275	RI	−0.065	0.215
EF	0.045	0.390			
SV	0.035	0.506	BA		
CO	0.175	0.001**	V_m_	0.119	0.023*
Tricuspid regurgitation	0.044	0.400	V_s_	0.077	0.141
			V_d_	0.121	0.021*
Cerebral hemodynamics			PI	−0.105	0.045*
Left side MCA			RI	−0.107	0.042*
V_m_	0.033	0.536			
V_s_	0.018	0.733	Asymmetry of VAs		
V_d_	0.039	0.454	ΔV_s_	0.080	0.130
PI	−0.039	0.458	ΔV_d_	0.137	0.009**
RI	−0.050	0.345	ΔV_m_	0.105	0.046*
			AI%	0.106	0.043*
Left side VA					
V_m_	0.120	0.022*	Conduction		
V_s_	0.067	0.203	CVCi	0.019	0.716
V_d_	0.157	0.003**			

*p is 0.05 or less, **p is 0.01 or less

HAH was positively associated with HR, CO and cerebral velocities while it negatively correlated with SpO_2_, PIs and RIs in BA and left side VA

### Risk factors for HAH at 3700 m

Univariate logistic regression was performed for variable with a p < 0.05 in correlation analyses or significant difference between HAH+ and HAH- groups. HR, SpO_2_, CO, V_d_, PI and RI of left side VA, velocities in BA (V_m_, V_d_) and ΔV_d_ were risk factors for HAH. After adjusted regression analyses, only HR (or CO) and ΔV_d_ were independent risk factors for HAH (Table 4).

**Table 4:** Univariate and adjusted logistic regression for variables at 3700 m (N = 363)

	Risk factors	β-coefficient	Odds ratio	(95% CI)		p value
				Lower	Upper	
Univariate logistic regression						
	HR	0.022	1.022	1.003	1.042	0.027*
	SpO_2_	−0.081	0.922	0.851	1.000	0.049*
	CO	0.422	1.524	1.195	1.944	0.001**
Left VA	V_d_	0.052	1.053	1.001	1.108	0.046*
	PI	−1.562	0.210	0.047	0.944	0.042*
	RI	−4.476	0.011	0.000	0.591	0.026*
BA	V_m_	0.032	1.033	1.005	1.061	0.018*
	V_d_	0.044	1.036	1.001	1.071	0.035*
Asymmetry of VAs	ΔV_d_	0.063	1.065	1.011	1.122	0.018*
Adjusted logistic regression						
Model 1						
	HR	0.025	1.025	1.005	1.045	0.012*
Model 2						
	HR	0.023	1.023	1.003	1.044	0.026*
	ΔV_d_	0.065	1.068	1.013	1.126	0.015*
Model3						
	CO	0.417	1.517	1.189	1.937	0.001**
	ΔV_d_	0.063	1.065	1.009	1.124	0.022*

*p is 0.05 or less, **p is 0.01 or less

Model 1: Adjusted by primary variables (SpO_2_, V_d_ in left side VA and BA);

Model 2: Adjusted by primary variables and an asymmetry data (ΔV_d_);

Model 3: Avoided the colinearity between HR and CO, indicated that CO and ΔV_d_ were independent risk factors for HAH;

We just listed the variable with a p < 0.05

### Associations between hemodynamic parameters and HAH in a subgroup of 129 subjects

In the baseline parameters, CO, V_m_, PI and RI in left side VA, velocities [V_m_ (60.20 ± 10.37 vs. 54.29 ± 7.03 cm/s, p = 0.004), V_s_ (92.11 ± 14.53 vs. 85.64 ± 10.46 cm/s, p = 0.023) and V_d_ (41.42 ± 7.60 vs. 37.19 ± 5.50 cm/s, p = 0.005)] in right MCA were significant different between HAH+ and HAH- groups. Furthermore, CO showed a positive relationship with HAH (Table 5).

**Table 5:** Associations between baseline parameters and HAH (N = 129)

	Differences between HAH+			Correlation with HAH intensities	
	and HAH- groups				
Headache Cases(n)	HAH-(31)	HAH+(98)	p value	r	p value
Demographic factors					
Age	22.71 ± 3.21	23.13 ± 4.31	0.616	0.093	0.297
BMI	21.41 ± 1.67	22.07 ± 2.52	0.175	0.153	0.084
Smoking(yes)	7(22.5%)	23(23.5%)	0.919	/	/
Alcohol consumption(yes)	21(67.7%)	58(59.2%)	0.396	/	/
Primary headache(yes)	4(12.9%)	23(23.5%)	0.209	/	/
Systemic hemodynamic					
SBP	118.35 ± 10.81	117.32 ± 12.37	0.676	0.018	0.843
DBP	74.00 ± 9.22	75.18 ± 11.31	0.598	0.059	0.509
ΔBP	44.35 ± 6.47	42.13 ± 6.37	0.094	−0.137	0.122
MAP	88.78 ± 9.29	89.22 ± 11.28	0.843	0.061	0.490
HR	63.32 ± 11.35	64.42 ± 10.10	0.607	0.008	0.930
SpO2	98.35 ± 1.02	98.30 ± 1.10	0.793	0.038	0.670
LA	31.06 ± 1.55	30.87 ± 1.78	0.581	−0.062	0.484
LV	45.81 ± 2.14	46.71 ± 2.50	0.071	0.183	0.037*
RA	35.45 ± 1.98	35.78 ± 2.27	0.478	0.153	0.082
RV	35.29 ± 1.85	35.55 ± 2.13	0.542	0.109	0.217
PA	20.06 ± 1.46	20.30 ± 1.63	0.481	0.094	0.288
EF	62.90 ± 5.98	63.10 ± 5.34	0.865	0.002	0.978
SV	62.53 ± 16.31	66.55 ± 10.61	0.113	0.088	0.322
CO	3.79 ± 1.01	4.33 ± 0.93	0.007**	0.177	0.045*
Tricuspid regurgitation	0.50(1.00)	0.45(1.00)	0.372	−0.030	0.739
Cerebral hemodynamic					
Left side MCA					
V_m_	59.61 ± 8.24	63.24 ± 10.92	0.091	0.060	0.501
V_s_	93.48 ± 12.13	97.00 ± 15.21	0.246	0.062	0.488
V_d_	40.93 ± 6.20	43.62 ± 8.37	0.102	0.058	0.513
PI	0.889 ± 0.154	0.852 ± 0.125	0.180	−0.004	0.960
RI	0.562 ± 0.051	0.550 ± 0.047	0.221	−0.015	0.862
Left side VA					
V_m_	30.94 ± 5.25	33.23 ± 4.97	0.029*	0.171	0.053
V_s_	49.64 ± 7.11	50.82 ± 7.56	0.444	0.083	0.351
V_d_	20.68 ± 4.17	22.26 ± 3.95	0.056	0.142	0.107
PI	0.957 ± 0.193	0.873 ± 0.169	0.022*	−0.150	0.090
RI	0.594 ± 0.075	0.561 ± 0.056	0.010*	−0.159	0.072
Right side MCA					
V_m_	54.29 ± 7.03	60.20 ± 10.37	0.004**	0.155	0.080
V_s_	85.64 ± 10.46	92.11 ± 14.53	0.023*	0.125	0.157
V_d_	37.19 ± 5.50	41.42 ± 7.60	0.005**	0.114	0.200
PI	0.888 ± 0.150	0.855 ± 0.146	0.276	−0.078	0.380
RI	0.574 ± 0.062	0.586 ± 0.053	0.592	−0.093	0.292
Right side VA					
V_m_	32.77 ± 5.30	34.84 ± 4.40	0.032	0.126	0.155
V_s_	49.38 ± 6.88	51.03 ± 6.33	0.220	0.073	0.411
V_d_	22.09 ± 4.29	23.64 ± 3.95	0.065	0.110	0.216
PI	0.849 ± 0.160	0.793 ± 0.156	0.086	−0.141	0.110
RI	0.555 ± 0.055	0.534 ± 0.061	0.081	−0.134	0.130
BA					
V_m_	37.55 ± 7.36	40.50 ± 7.80	0.065	0.154	0.082
V_s_	66.29 ± 13.65	68.53 ± 13.87	0.433	0.054	0.546
V_d_	23.48 ± 5.08	25.69 ± 5.97	0.066	0.139	0.115
PI	0.964 ± 0.210	0.885 ± 0.197	0.092	−0.104	0.239
RI	0.644 ± 0.055	0.621 ± .0714	0.108	−0.074	0.404
Asymmetry of bilateral VAs					
ΔV_s_	2.0 (15.0)	0.0(9.25)	0.795	0.042	0.634
ΔV_d_	0.0(6.0)	−2.0 (6.0)	0.703	0.017	0.851
ΔV_m_	−2.0 (8.0)	−1.0 (7.0)	0.934	0.064	0.468
AI%	−0.061 (0.224)	−0.034(0.200)	0.815	0.075	0.398
Conduction					
CVCi	0.648 ± 0.103	0.704 ± 0.145	0.048*	0.097	0.275

/:binary variables that were not analyzed by Spearman’s correlation

*p is 0.05 or less, **p is 0.01 or less

Univariate logistic regression revealed that CO, V_m_ in both VAs and velocities in right side MCA were risk factors for HAH. However, adjusted analyses showed that CO (adjusted by V_s_ and V_d_ of right side MCA, OR: 1.613, p = 0.036 or adjusted by V_m_ of right side MCA OR: 1.811, p = 0.024) and V_m_ in right MCA (OR: 1.066, p = 0.011) or left VA (OR: 1.099, p = 0.035) were still independent predictors for HAH (Table 6).

**Table 6:** Univariate and adjusted logistic regression for variables at baseline to predict HAH. (N = 129)

	Predictors	β-coefficient	Odds ratio	(95% CI)		P value
				Lower	Upper	
Univariate logistic regression						
	CO	0.650	1.915	1.158	3.168	0.011*
Left side VA	V_m_	0.095	1.099	1.008	1.198	0.031*
Right side MCA	V_m_	0.071	1.073	1.021	1.127	0.005**
	V_s_	0.039	1.040	1.005	1.076	0.026*
	V_d_	0.087	1.091	1.024	1.161	0.007**
Right side VA	V_m_	0.099	1.104	1.007	1.211	0.035*
Adjusted logistic regression						
Model 1						
Left VA	V_m_	0.094	1.099	1.007	1.199	0.035*
	CO	0.478	1.613	1.031	2.522	0.036*
Model 2						
Right side MCA	V_m_	0.064	1.066	1.015	1.121	0.011*
	CO	0.594	1.811	1.080	3.036	0.024*

Model 1: Adjusted by primary variables (V_s_ and V_d_ of right side MCA)

Model 2: Adjusted by calculated variable (V_m_ of right side MCA)

Baseline CO was an independent predictor for HAH at 3700 m, whereas, V_m_ in left VA or right MCA was also independent predictors for HAH in different models

Only the variable with a significant statistically has been listed above

* p is 0.05 or less, ** p is 0.01 or less

Furthermore, in the subgroup analysis, the change in tricuspid regurgitation from sea level to 3700 m was significantly different between the HAH+ and HAH- groups. The alterations of ΔV in V_d_ and Vs of the VAs and the V_m_ and V_d_ of the BA were also significantly different between the HAH+ and HAH- groups (see Additional file 1: Table S1A).

## Discussion

### Alterations in both systemic and cerebral hemodynamics

In the current study, we identified alterations in both systemic and cerebral hemodynamics. The HR dramatically increased between sea level and high altitudes, which is consistent with a previous study that reported a significant increase in HR after exposure to high altitude because of the activation of the parasympathetic nervous system by hypobaric hypoxic stress [15, 4, 16]. However, the SV was similar at both altitudes, which indicates the increased CO was primarily caused by the increased HR [16]. Conversely, the hypoxia also induced a reduction in the SpO_2_, which may result in a decrease in the delivery of oxygen and energy to organs and tissues. Furthermore, the diastolic function of the heart has been reported to decrease in high altitude environments, which may be because of the shortened cardiac diastolic phase caused by the increased HR [17, 16].

Following acute hypoxic stress, parasympathetic nervous system activation, combined with a disequilibrium in the synthesis and expression of certain vasoconstrictors and vasodilators, results in endothelial cell dysfunction (e.g., increased endothelin-1 expression vs. decreased nitric oxide level) [18, 19]. Therefore, the vasoconstriction of systemic and cerebral arteries exhibits a significant impact on the measured velocities of blood flow [20]. We demonstrated that most velocities in both the anterior and posterior cerebral circulations significantly increased. These results are similar to previous studies in which cerebral blood flow increased dramatically after high altitude exposure [21–23].

### Associations between HAH occurrence and demographical data

As previously reported by our team and other researchers, HAH patients are typically older in age, which is consistent with the risk factors of primary headache at sea level and high altitude [8, 9]. Although a history of smoking and alcohol consumption did not exhibit associations with HAH, they were not precisely measured, which could be improved in future studies.

### HAH is partially associated with systemic hemodynamics

HAH is a regional symptom that comprises the primary diagnostic criteria of acute mountain sickness, which is typically induced by a local and systematic factor [5, 24]. Therefore, systemic hemodynamics may be a cause of HAH because it functions to deliver blood to all organs, including the brain, which is an organ with considerably high metabolism and energy consumption. First, an increased HR exhibited a strongly positive correlation with HAH, which is consistent with the relationship between CO and HAH. The HAH patients were also characterized by increased HR and CO. Further analyses indicated that baseline CO and LV were correlated with HAH significantly. These results may be because of the nervous system changes that lead to alterations in systemic hemodynamics [19, 25, 16]. In addition, the SpO_2_ was inversely correlated with HAH; however, the SBP and DBP were not related to HAH, which was similar to previous studies [8, 15, 25]. A reduced SpO_2_ and increased HR are independent risk factors for HAH [8, 9]. These changes could result from sympathetic nervous system activation and a decreased partial pressure of oxygen, which results in a decrease in oxygen delivery to the brain, where oxygen consumption is increased during hypoxic stress [5, 9, 6]. The change in tricuspid regurgitation in HAH patients differed from the non-HAH subjects, which indicates alterations in cardiac function induced by hypoxia may be risk factors for HAH (see Additional file 1).

### Cerebral hemodynamic characteristics of HAH

In the present study, we demonstrated that the velocities in both the BA and left VA in the posterior cerebral circulation were strongly associated with HAH, which is partly consistent with previous reports [26, 22, 27]. The HAH patients were characterized by an increased V_d_ but decreased RI and PI in the left VA. These findings imply that HAH is associated with higher blood flow but lower resistance and compliance. At the baseline, HAH+ patients were also featured with a higher V_m_ in VAs on both sides indicating that posterior circulation plays critical roles in HAH or it is a pathophysiological process of HAH. Furthermore, the HAH+ group also exhibited increased V_s_ and V_d_ in the BA, which supplies blood and oxygen to the cerebellum. These results are consistent with the clinical characteristics that HAH often presents (i.e., a pulsing headache), which may be caused by diastolic dysfunction in the arteries [5, 6]. The relationship between HAH and posterior, rather than anterior, cerebral circulation contributed to HAH occurrence.

Of note, velocities in right MCA at baseline in HAH+ group were differed significantly from those in HAH- group. In partial agreement with the results reported for acute mountain sickness, including HAH, we did not identify an association between HAH and anterior circulation as assessed by the velocities, PI and RI of the bilateral MCAs [28]. However, these results were inconsistent with the previously reported that acute mountain sickness related headache usually presents as frontal headache [5]. The inconsistence may be caused by the sample size in the subgroup of 129 participants.

Though the baseline conduction from systemic to cerebral blood flow, which was evaluated using the cerebrovascular conductance index, showed a significant difference between HAH+ and HAH- groups, it is not predictor for HAH. Furthermore, it was not associated with HAH at 3700 m, which indicates cerebral blood flow plays a primary role in HAH and has more critical importance than systemic hemodynamics.

#### Predictors and risk factors for HAH

After adjusted regression analyses at baseline, we identified that CO and V_m_ in left VA (or right MCA in different regressions) were independent predictors for HAH. However, the posterior and anterior circulations were not valuable in a same model.

Other adjusted analyses at 3700 m revealed that only HR (or CO) and ΔV_d_ were independent risk factors for HAH, which is partly consistent with our and others’ previous studies. However, ΔV_d_ was found to be a novel risk factor for HAH which may be caused by the hypoxia induced local expressions of vasoactive substances. Furthermore, the variation in VAs of Chinese Han in anatomy may also be a reason for our findings.

### Relationship between HAH and VA asymmetry

We report a novel finding in that VA asymmetry was closely related to HAH. An asymmetry in the bilateral isonym VA exists under normal physiological conditions [21, 28, 29]. However, this physiological asymmetry could become a considerable or pathological irregularity because of its amplification by hypoxia-induced vasodilatation and an impaired auto-regulation of cerebral blood flow following acute high-altitude exposure [21].

Furthermore, HAH severity was closely associated with the VA asymmetry, which represents a characteristic that has not been previously reported. This finding may be associated with the region of blood flow and perfusion in the cortex and cerebellum. It may also be related to the clinical characteristics of the migraine pattern present in many HAH patients [5]. The variance between the isonym VAs may contribute to the development of HAH and requires further examination to identify its precise pathophysiological mechanisms.

HAH comprises multifactorial components [30, 12, 31, 14]. Therefore, the cerebral and systemic hemodynamic characteristics of HAH may be relevant to the disease development, as well as its pathophysiological processes and clinical manifestations.

### Limitations

Our study was restricted to young Chinese men, which could potentially generate a bias regarding age or gender; thus, this issue should be addressed in future studies. Another limitation is that a key modulator of cerebral perfusion, partial pressure of carbon dioxide (PaCO_2_), was not measured in the present study. This modulator could be included in future mechanistic studies. Another limitation is that headache satisfied the diagnostic criteria of the International Classification of Headache Disorders basically. Although the descent was not performed immediately after the onset of the headache, cephalalgia almost resolved as time went on. Furthermore, the subjects were all newcomers from sea level, thus none of them with a history of HAH or acute mountain sickness history, which should be improved in future study.

## Conclusions

HAH is characterized, in part, by increased systemic hemodynamics and posterior cerebral circulation as reflected by the velocities in the BA and left VA, as well as lower arterial resistance and compliance. Furthermore, baseline CO and V_m_ in left VA or right MCA at sea level are independent predictors for HAH, whilst bilateral VA asymmetry may contribute to the development of HAH at high-altitude.

## Additional file

Additional file 1: Table S1A. Associations between changes of hemodymics and HAH in the subgroup.

## Abbreviations

BA: Basilar artery
CO: Cardiac output
DBP: Diastolic blood pressure
EF: Ejection fraction
HAH: High-altitude headache
HAH+: HAH positive or with HAH
HAH-: HAH negative or without HAH
HR: Heart rate
LA: End-diastolic internal diameters of left atrium
LV: End-diastolic internal diameters of left ventricle
MCA: Middle cerebral artery
PA: End-diastolic internal diameters of pulmonary artery
PI: Pulsatility index
RA: End-diastolic internal diameters of right atrium
RI: Resistance index
RV: End-diastolic internal diameters of right ventricle
SBP: Systolic blood pressure
SpO_2_: Saturation pulse oxygen
SV: Stroke volume
VA: Vertebral artery
V_d_: Diastolic velocity
V_m_: Mean velocity
V_s_: Systolic velocity
ΔV_d_: Difference in diastolic velocity between bilateral isonym cerebral arteries
ΔV_m_: Difference in mean velocity between bilateral isonym cerebral arteries
ΔV_s_: Difference in systolic velocity between bilateral isonym cerebral arteries
