# Supplementary material for: Hemodynamic characteristics of high-altitude headache following acute high altitude exposure at 3700 m in young Chinese men
Source: J Headache Pain. 2015 May 12;16:43. doi: 10.1186/s10194-015-0527-3 (PMC4431987; doi:10.1186/s10194-015-0527-3)
Supplement: Additional file 1: Table S1A. — Associations between changes of hemodymics and HAH in the subgroup. [file 10194_2015_527_MOESM1_ESM.doc]

Table 1A. Associations between changes of hemodymics and HAH in the subgroup

|  | Differences between HAH+ and HAH- groups | | | Correlation with HAH intensities | |
| --- | --- | --- | --- | --- | --- |
| Alterations | HAH+(98) | HAH-(31) | p# | r | p# |
| Tricuspid regurgitation | 0.050(1.00) | -0.200(0.50) | 0.030* | 0.116 | 0.189 |
| V_m_ of BA | 3.00(10.25) | -1.00(7.50) | 0.011* | 0.203 | 0.021* |
| V_d_ of BA | 3.00(8.00) | 2.00(7.00) | 0.014* | 0.215 | 0.014* |
| ΔV_s_ of VA | 0.00(11.00) | -3.00(9.50) | 0.050* | 0.164 | 0.063 |
| ΔV_d_ of VA | 1.00(7.25) | -2.00(4.50) | 0.029* | 0.189 | 0.032* |
| HR | 17.0(21.0) | 20.0(17.5) | 0.256 | 0.182 | 0.039* |

Only variable with a p < 0.05 in either of the two analyses have been listed above.

# in the subgroup of 129 volunteers

* p is 0.05 or less, ** p is 0.01 or less.

HAH was also associated with changes in tricuspid regurgitation BA velocities and VAs asymmetries.

In the subgroup analyses, although tricuspid regurgitation was not associated with HAH at high altitudes, the change in tricuspid regurgitation from sea level to 3700 m was significantly different between the HAH+ and HAH- groups in the 129 subjects (see additional file). The alterations of ΔV in V_d_ and V_s_ of the VAs and the V_m_ and V_d_ of the BA were also significantly different between the HAH+ and HAH- groups (see additional file).
